# Supplementary material for: Splice-Junction-Based Mapping of Alternative Isoforms in the Human Proteome
Source: Cell Rep. Author manuscript; Available in PMC 2020 Jan 15. (PMC6961840; doi:10.1016/j.celrep.2019.11.026)
Supplement: 2 [file NIHMS1546469-supplement-2.zip › DF1/PXD010154/Esophagus/Esophagus_1_SVIL_RPELCTSHSETPTVDDEEKVDER.pdf]

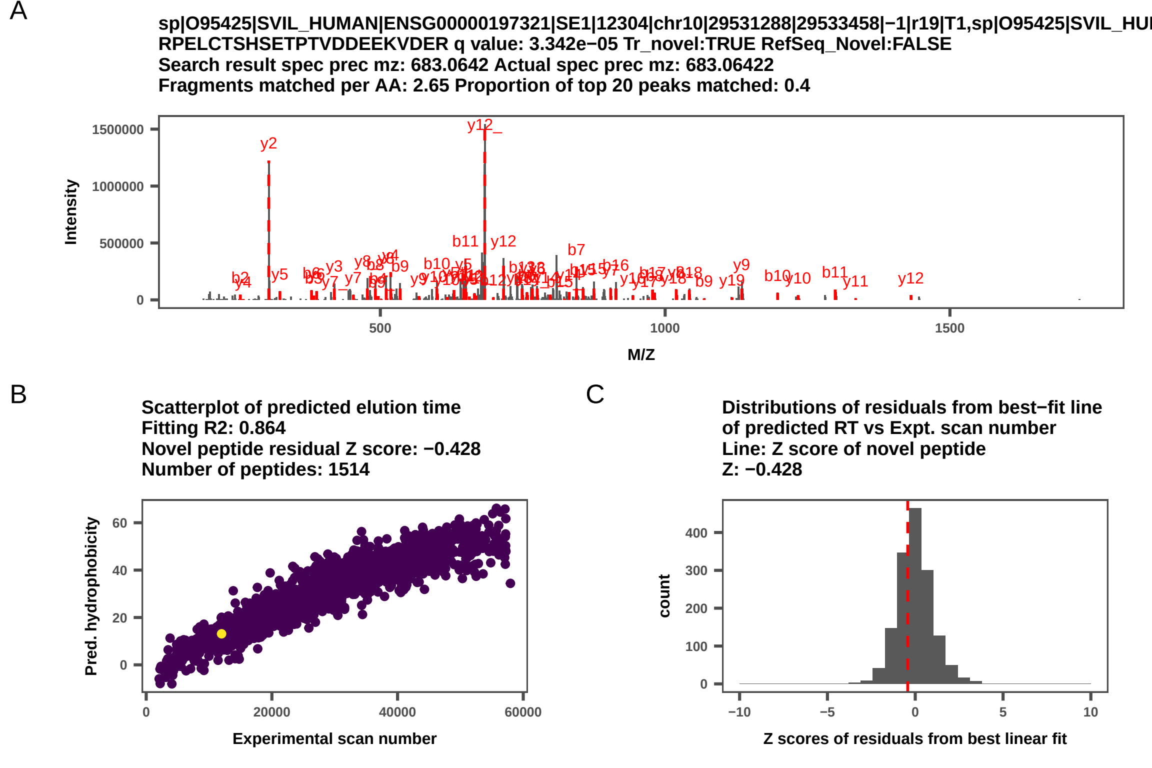
A
sp|O95425|SVIL_HUMAN|ENSG00000197321|SE1|12304|chr10|29531288|29533458|−1|r19|T1,sp|O95425|SVIL_HU
RPELCTSHSETPTVDDEEKVDER q value: 3.342e−05 Tr_novel:TRUE RefSeq_Novel:FALSE
Search result spec prec mz: 683.0642 Actual spec prec mz: 683.06422
Fragments matched per AA: 2.65 Proportion of top 20 peaks matched: 0.4
Intensity
1500000
1000000
500000
0
500
1000
1500
M/Z
B
Scatterplot of predicted elution time
Fitting R2: 0.864
Novel peptide residual Z score: −0.428
Number of peptides: 1514
Pred. hydrophobicity
60
40
20
0
0
20000
40000
60000
Experimental scan number
C
Distributions of residuals from best−fit line
of predicted RT vs Expt. scan number
Line: Z score of novel peptide
Z: −0.428
count
400
300
200
100
0
−10
−5
0
5
10
Z scores of residuals from best linear fit
